# Conebeam CT‐guided 3D MLC‐based spatially fractionated radiation therapy for bulky masses

**DOI:** 10.1002/acm2.13608

**Published:** 2022-04-21

**Authors:** Damodar Pokhrel, Mark E Bernard, Richard Mallory, William St Clair, Mahesh Kudrimoti

**Affiliations:** ^1^ Department of Radiation Medicine, University of Kentucky Lexington Kentucky USA

**Keywords:** bulky tumors, dose‐escalation, GRID‐block, MLC, SFRT, toxicity‐reduction

## Abstract

For fast, safe, and effective management of large and bulky (≥8 cm) non‐resectable tumors, we have developed a conebeam CT‐guided three‐dimensional (3D)‐conformal MLC‐based spatially fractionated radiation therapy (SFRT) treatment. Using an in‐house MLC‐fitting algorithm, Millennium 120 leaves were fitted to the gross tumor volume (GTV) generating 1‐cm diameter holes at 2‐cm center‐to‐center distance at isocenter. SFRT plans of 15 Gy were generated using four to six coplanar crossfire gantry angles 60° apart with a 90° collimator, differentially weighted with 6‐ or 10‐MV beams. A dose was calculated using AcurosXB algorithm, generating sieve‐like dose channels without post‐processing the physician‐drawn GTV contour within an hour of CT simulation allowing for the same day treatment. In total, 50 extracranial patients have been planned and treated using this method, comprising multiple treatment sites. This novel MLC‐fitting algorithm provided excellent dose parameters with mean GTV (V7.5 Gy) and mean GTV doses of 53.2% and 7.9 Gy, respectively, for 15 Gy plans. Average peak‐to‐valley dose ratio was 3.2. Mean beam‐on time was 3.32 min, and treatment time, including patient setup and CBCT to beam‐off, was within 15 min. Average 3D couch correction from original skin‐markers was <1.0 cm. 3D MLC‐based SFRT plans enhanced target dose for bulky masses, including deep‐seated large tumors while protecting skin and adjacent critical organs. Additionally, it provides the same day, safe, effective, and convenient treatment by eliminating the risk to therapists and patients from heavy gantry‐mounted physical GRID‐block—we recommend other centers to use this simple and clinically useful method. This rapid SFRT planning technique is easily adoptable in any radiation oncology clinic by eliminating the need for plan optimization and patient‐specific quality assurance times while providing dosimetry information in the treatment planning system. This potentially allows for dose‐escalation to deep‐seated masses to debulk unresectable large tumors providing an option for neoadjuvant treatment. An outcome study of clinical trial is underway.

## 1 INTRODUCTION

Radiation therapy treatment for patients with advanced bulky tumors (≥8 cm) for palliative care or curative intent via spatially fractionated radiation therapy (SFRT), also known as GRID therapy, started in the orthovoltage Era with a grid collimator.^1^ Utilizing a physical Cerrobend GRID‐block can allow a regrowth of skin and subcutaneous tissue under the blocked area and manage normal tissue toxicity. In the megavoltage Era, the management of unresectable bulky tumors using an open high‐energy X‐ray field has been converted to a set of pencil beam–type radiation fields using an external physical GRID‐block comprising brass, cerrobend, or lead in order to deliver a large single dose of 10–20 Gy.^2^, ^3^, ^4^ This large dose can be followed by a conventional radiotherapy treatment of 30–70 Gy, concurrent chemoradiation, or neoadjuvent surgical resection. GRID therapy has resulted in a significant tumor reduction (62%–91%) with a single dose of 15 Gy or higher followed by the conventional radiotherapy with a 78% pain relief response rate, 20% complete clinical response, and 73% rates of mass effect with and without conventional radiotherapy treatment in the curative setting and maintaining the skin toxicity. Typically, sarcoma and head‐and‐neck cancer responded very well.^2^, ^3^, ^4^, ^5^, ^6^ The underlying mechanisms of the SFRT treatment response have not been fully explored yet. However, there are several speculative theories to contribute the promising clinical outcomes of SFRT therapy. The first is radiation‐induced bystander effect^7^, ^8^ that is associated with the induction of radiation effects in low‐dose locations adjacent to hit tumor cells via cell signaling. For this reason, this effect is stronger in high‐dose gradient—characteristics of the peak‐to‐valley dose ratio (PVDR) with a 50:50 open‐to‐closed‐area of the Cerebobd GRID‐block. A typical reported PVDR estimates from 3 to 5. Second, in addition to direct cell‐kill (DNA double‐strand breaks), it provides damage to the intratumor microvasculature structure by eliminating immature and weak tumor vessels that are irregularly dilated, constricted, and branched. These tumor blood vessels are rather fragile, disrupted, and susceptible to a high single dose that leads to indirect cell death. Third, increasing antitumor immune response by ablative high doses of radiation will upregulate the various immunostimulatory cytokines, which then interacts with the tumor antigens released from the dying tumor cells, thereby provoking an antitumor immune response in weeks or months.^9^, ^10^ All these mechanisms can contribute to great treatment response that is clinically seen by a dramatic regression of large tumor masses. Unlike conformal radiation therapy, only a fraction of tumor volume is irradiated by primary beam, and other fraction receives only scattered radiation, therefore enhancing bystander effect to the tumor mass as described previously, and also enabling sparing adjacent critical organs and reducing skin oxicity.^11^, ^12^

However, there are major limitations of the conventional physical single‐field GRID‐block therapy: (1) deep‐seated bulky tumors may only receive a third or less of the prescribed dose of 15 Gy. (2) It is difficult to manage skin toxicity, while escalating the tumor dose, and difficult to spare immediately adjacent critical organs. (3) The physical GRID‐block is not readily available to any standard radiation therapy clinics. (4) Lifting a very heavy physical GRID‐block (about 25 lb) poses a serious safety concern to radiation therapy staff and for cancer patient at certain gantry angles. (5) Due to the lack of commissioning of the physical GRID‐block in the treatment planning system (TPS), the dosimetric details of isodose distribution and dose–volume histogram parameters prediction are not always available in the user's TPS for the documentation of dose to critical organs for physician plan review. However, in the modern Era, there are advanced treatment planning and delivery approaches for the management of large and bulky tumors using SFRT, including a single‐field step‐and‐shoot MLC plan or a fully optimized IMRT, VMAT, or helical tomotherapy plans.^13^, ^14^, ^15^, ^16^ Moreover, the robotic CyberKnife, microbream, or proton GRID therapy can be offered to the select SFRT patients.^17^, ^18^, ^19^ However, these modern SFRT approaches may not be accessible for the same day or next day of SFRT due to the (1) need for a third‐party software to generate a three‐dimensional (3D) lattice structure of the GRID contour for plan optimization, (2) required longer treatment planning time for IMRT, VMAT, or inversely optimized tomotherapy plan, at least for a few days, (3) need for extensive physics quality assurance and second checks, (4) much longer treatment delivery time, and at least 20–60 min could potentially increase intrafraction motion error, patient inconvenience, and potentially slowing down clinic workflow; and (5) highly modulated MLC or tomotherapy plan leads to higher leakage and transmission dose; all in all contributing for hindering of the same day or next day SFRT treatment in the standard radiation therapy center.^13^, ^14^, ^15^, ^16^, ^17^, ^18^, ^19^ Moreover, not every candidate of GRID therapy patients can have access to rare and expensive treatment modalities such as robotic CyberKnife or proton GRID treatment.^17^, ^18^, ^19^ To overcome the previously mentioned difficulties and address the immediate need for same or next day treatment of large and bulky tumors, we have further improved our previously reported 3D‐conformal MLC‐based forward planning and treatment delivery method^20^ for SFRT patients.

## 2 METHODS AND MATERIALS

In our previous prototype publication, for each patient, a physician drawn gross tumor volume (GTV) GRID contour was post‐processed to generate a 10‐mm diameter, and 20‐mm center‐to‐center distance grid‐pattern, similar to 3D‐lattice structure (inside the physician's drawn original contour) mimicking the conventional GRID‐block design using an in‐house MATLAB program.^20^ The standalone program read the 3D‐CT images and structure set (GTV contour) in DICOM format. A voxel mask of the GRID‐lattice structure was created inside the GTV structure using MATLAB's boundaries function in DICOM format. The 3D‐lattice structure was then imported back into the Eclipse TPS for 3D‐MLC‐based crossfire forward planning. However, in daily clinical practice, it was realized that utilizing the standalone third‐party MATLAB code to generate a 3D‐lattice structure was impractical due to additional time exporting and importing planning CT images and structure datasets. Moreover, extra time generating and reviewing this new contour is needed in a standalone MATLAB script. Lastly, a dedicated expert planner in the clinic is required, which we found unfavorable for implementing this simple and clinically useful approach in the standard radiotherapy center, including academic and community centers. Therefore, to overcome the major limitation, for those select gantry angles and collimator setting (similar beam geometry as before), we have further developed this method of MLC‐fitting algorithm in the Eclipse TPS that do not need post‐processing of the physician drawn GTV GRID contour. It simply allows for generating the clinical SFRT plan via 3D‐MLC fitting to the GTV target in the TPS—avoiding the major concern of using third‐party software to generate, re‐review, exporting, and importing a 3D lattice structure in the TPS for SFRT planning.

**FIGURE 1:**
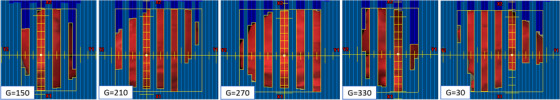
Illustration of the in‐house 3D‐MLC‐fitting algorithm to the physician‐delineated GRID GTV target (red) without post‐processing the GTV contour. Five of six gantry angles were used to treat large and bulky (501 cm^3^, 10‐cm diameter) right pelvis mass for 15 Gy in one fraction. Some peripheral MLCs positions were adjusted to further minimize the dose to immediately adjacent critical organ, including small bowel. GTV, gross tumor volume

Additional improvement and refinement of this approach utilized an in‐house MLC‐fitting algorithm in Eclipse TPS^21^, ^22^, ^23^ using the 120 Millennium MLC leaves to GTV to generate 1‐cm diameter holes and 2‐cm center‐to‐center distance (at isocenter) without generating a 3D‐lattice structure. Briefly, for a single high‐dose of 15 Gy, 3D MLC‐based SFRT plans can be generated using six coplanar crossfire gantry angles at 60° equal‐spacing (30°, 90°, 150°, 210°, 270°, and 330°) with 90° collimator setting of each treatment field for the differentially weighted 6‐ or 10‐MV photon beams and Advanced Acuros XB dose calculation engine that generates a brachytherapy sieve‐like dose channeling without post‐processing the physician‐drawn GTV contour, similar to previously reported beam geometry. Similar to conventional 3D‐conformal radiotherapy planning, beam weighting factors for each gantry position were adjusted based on tumor location, tumor depth, and proximity of the critical organs, with the intent of optimizing the target coverage and minimizing the maximal dose to the immediately adjacent critical organs following the single treatment SBRT guidelines.^24^ However, unlike our previous approach of regenerating a 3D‐lattice GRID structure using offline software,^20^ the new semiautomated MLC‐fitting algorithm in Eclipse TPS is as follows:

1. For each select gantry angle, fit the MLC aperture to the physician drawn GTV GRID contour.
2. Open two MLCs (10 mm) at the isocenter (center of mass of GRID contour), and park these MLCs outside the jaws—eliminating the end leaf leakage.
3. Close two MLCs (10 mm) on both left and right sides of the target, followed by opening two MLCs (10 mm) as shown in Figure 1—generating 10‐mm hole sizes and 20‐mm center‐to‐center distance for each hole at isocenter, similar to the traditional physical cerrobend GRID‐block pattern.
4. For each treatment field, repeat this process until reaching the boundary of the physician‐drawn GRID contour all around the GTV target.
5. Differentially adjust beam‐weighting of each treatment field and beam energy, as needed.
6. Manually adjust peripheral individual MLC(s) leaf tip position to further reduce the dose to critical organs, if required.

**FIGURE 2:**
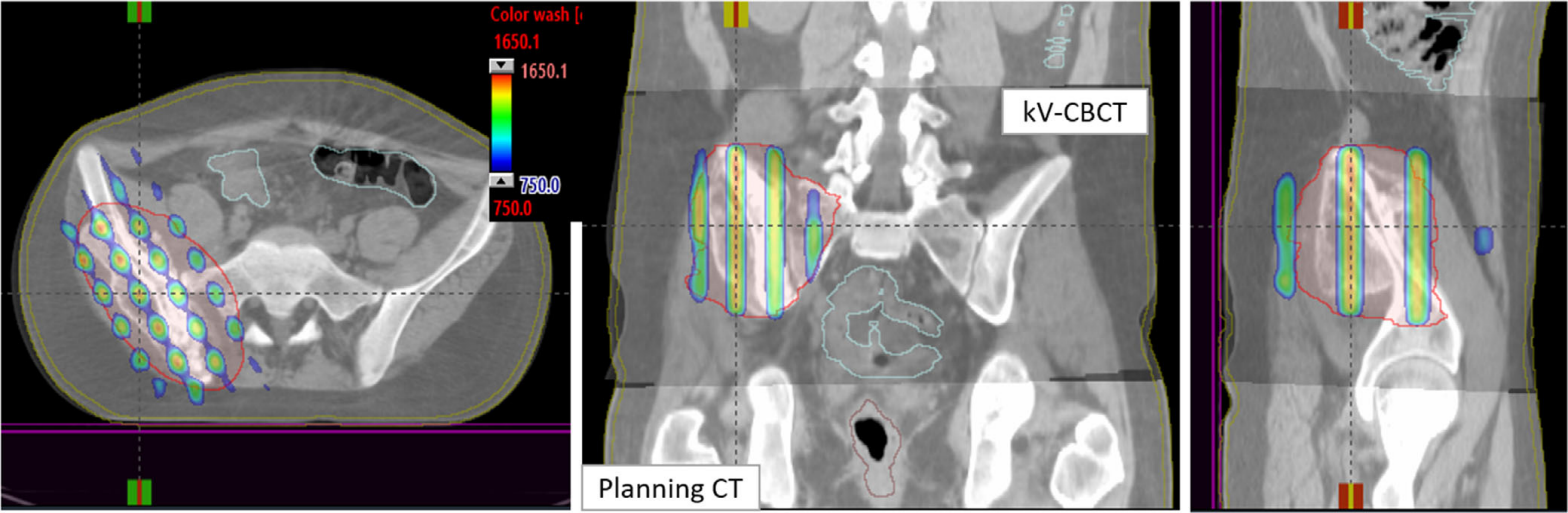
Demonstration of axial, coronal, and sagittal views of kV‐CBCT images (see the inset) coregistered with planning CT images (see the back of coronal and sagittal views) used for CBCT‐guided SFRT treatment on TrueBeam Linac. The overlaid planned isodose colorwash (50%–110%) with anatomical landmarks is shown for a patient treated with MLC‐based 3D‐conformal SFRT (15 Gy in one fraction) for a deep‐seated bulky mass of 10.0‐cm‐diameter tumor in a right pelvis—malignant neoplasma of connective and soft tissue of trunk. CBCT images were acquired in the treatment position followed by performing automatic rigid‐registration and manually fine‐tuning the registration for tumor soft‐tissue alignment before applying the couch correction. 3D, three‐dimensional; SFRT, spatially fractionated radiation therapy

This way, we automatically identified the corresponding matching MLC(s) on each opposing gantry pair to open and block the required GRID contour—simply generating a highly heterogenous sieve‐like dose tunneling inside the GTV for debulking large and bulky unresectable masses and potentially allowing for the same day treatment as CT simulation. Moreover, in this MLC‐fitting approach, to minimize the MLC tip leakage and transmission as mentioned previously, the fitted MLCs from either MLC‐bank (A or B) were parked outside the jaws as shown in Figure 1, and the resulting isodose colorwash distribution is shown in Figure 2. Utilizing this novel MLC‐fitting algorithm, in the past 2 years, under the IRB approved protocol from our institution, we have planned and treated more than 50 extracranial patients (head‐and‐neck tumors, neglected breast masses, chest tumors, abdominal/pelvis masses, liver tumors, adrenal masses, paraspinal masses, and extremities) with large and bulky tumors using the 3D‐conformal MLC‐based SFRT on the same day as CT simulation or the next day. Patients were immobilized either using the long head‐and‐neck mask (with their arms on the side) or a VacLoc (CIVCO system, Orange City, IA) bag (with their arms above the head) in the supine position. All planning computed tomography (CT) images were acquired on a GE Lightspeed 16 slice CT scanner (General Electric Medical Systems, Waukesha, WI). The 3D‐CT images were acquired with 512 × 512 pixels at 2.5‐mm slice thickness, and the patient's skin spots were marked. The 3D‐CT images were then imported from the Varian Eclipse TPS (Varian, Palo Alto, CA). The treating physician delineated the GTV in 3D‐CT images in Eclipse TPS, and patients were treated on either TrueBeam or 21EX Linac equipped with a CBCT onboard imager. The GTV size ranged from 8.5 to 14.7 cm. Dosimetric parameters reported included  PVDR = GTVD10% ÷ GTVD90%, GTV (V7.5 Gy), mean GTV dose, skin dose, and maximal dose to immediately adjacent critical organs. Based on our previous clinical experiences, the departmental guidelines for achieving PVDR, GTV(V7.5 Gy), mean GTV dose of 15 Gy prescription were greater than 3.0, 50%, and 7.5 Gy, respectively. Maximal dose tolerances to the adjacent critical organs were evaluated in compliance with single‐dose NRG‐RTOG‐0915.^24^ Treatment planning and delivery times and patient setup accuracy were recorded.

## 3 RESULTS AND DISCUSSION

Our MLC‐GRID plans showed clinically acceptable dose parameters with mean GTV(V7.5 Gy) and mean GTV doses of 15 Gy being 53.2% ± 4.5% (range 50.5%–64.1%) and 7.9 ± 1.1 Gy (range 7.3–9.1 Gy), respectively, following our physicians’ prescription requirements. Average PVDR was 3.2 ± 0.6 (range 2.8–4.5). Maximum and dose to 5 cm^3^ of skin were 10.1 ± 3.3 Gy (range 5.5–13.2 Gy) and 6.3 ± 2.5 Gy (range 1.2–9.8 Gy), respectively. Site‐specific maximal doses to immediately adjacent critical organs were as follows: spinal cord < 3.0 Gy, larynx < 4.5 Gy, humeral head < 5.1 Gy, femoral head < 5.5 Gy, small bowel < 5.2 Gy, mean lung dose < 3.5 Gy, on average. Planning time was typically an hour for an experienced planner. Mean beam‐on time was 3.32 ± 0.18 min (range 3.12–3.55 min). Timing gantry angles were arranged for efficient treatment delivery on per‐patient basis. Mean treatment time, including patient setup and conebeam CT imaging to beam‐off, was 11.48 ± 1.5 min (range 10.10–14.5 min). Average 3D vector couch correction from the original skin‐marker was 0.55 ± 0.48 cm (range 0.25–1.89 cm).

Furthermore, in this MLC‐fitting approach, to minimize the MLC tip leakage and transmission, fitted MLCs from either bank were parked outside the jaws as shown in Figure 1, corresponding isodose colorwash, superimposed with the planning CT images in the treatment position, is shown in Figure 2. The planned isodose colorwash is superimposed with the daily pretreatment CBCT images after the couch corrections were applied. This patient was initially positioned using external skin marks and in‐room lasers, followed by a full kV‐CBCT scan. In‐house SBRT/IGRT protocol was applied to coregister the pretreatment CBCT with the planning CT scans (see Figure 2). Image registration was performed automatically based on region of interest and bony landmarks. Registration was followed by a manual refinement of the soft‐tissue tumor matching and confirmed by the attending physician before treatment. In this case, pretreatment IGRT couch correction was less than 5 mm in each direction for 3D MLC‐based SFRT treatment. For this patient, the GTV (V7.5 Gy) = 56%. Mean GTV dose was 8.8 Gy. PVDR was 3.5. Maximal and dose to 5 cm^3^ of skin were 7.6 and 5.2 Gy (see Figure 2), all parameters meeting our physician's prescription order. Immediately adjacent critical structures were also spared: maximal dose to right femoral head (4.7 Gy) and bowel (4.9 Gy). All of these dosimetric parameters were in compliance with RTOG single high‐dose treatment schemata.^24^ In this case, five of six treatment fields with 10‐MV beam, with a maximal dose rate of 600 MU/min (due to very large patient separation, 90° gantry angle was not used) was used. A total of 2123 MU doses were delivered in less than 3.5 min of beam‐on time via CBCT‐guided SFRT treatment, similar to SBRT patient setup. Total treatment time (including patient setup, CBCT‐imaging, and verification) was <15 min. Moreover, a palliative conventional dose of 37.5 Gy in 15 fractions to the right pelvis area was delivered, after a single dose of 15‐Gy SFRT treatment, sequentially.

**FIGURE 3:**
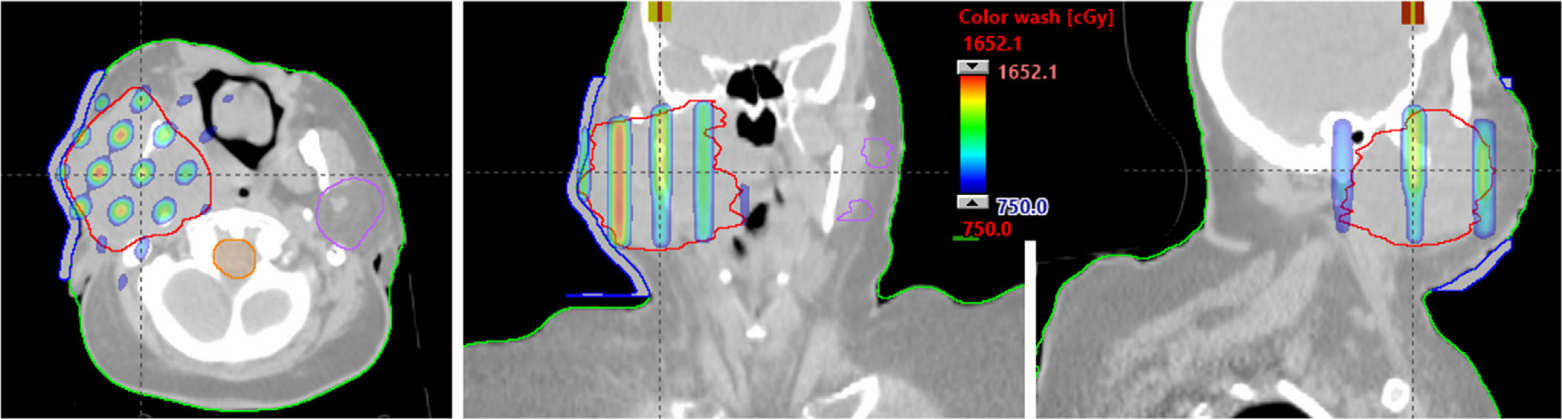
Axial, coronal, and sagittal views of an isodose colorwash of the MLC‐based 3D‐conformal SFRT plan in the treatment of Markel cell carcinoma of the large right neck mass (265 cm^3^, equivalent to a 8.4‐cm diameter) was treated for a single dose of 15 Gy, with a 110% hotspot inside the GTV. Immediately adjacent critical organs, such as spinal cord and brainstem, were spared. Moreover, to avoid contralateral parotid gland, 90° gantry angle was not used. 3D, three‐dimensional; GTV, gross tumor volume; SFRT, spatially fractionated radiation therapy

**FIGURE 4:**
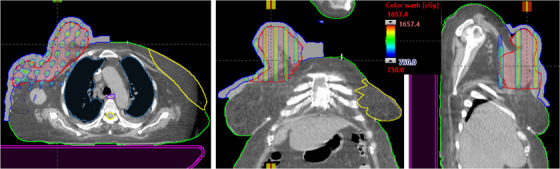
This is an example of the MLC‐based 3D‐conformal SFRT for the treatment of neglected female right breast patient of very large right breast mass. Due to the very large patient separation, 90° and 150° gantry angles were not used. Maximal dose to critical organs, such as spinal cord (1.9 Gy), left breast (1.6 Gy), heart (3.9 Gy), and mean lung dose (1.3) Gy, was achieved. 3D, three‐dimensional; SFRT, spatially fractionated radiation therapy

**FIGURE 5:**
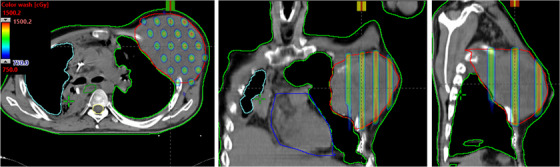
Demonstration of axial, coronal, and sagittal views of isodose colorwash (50%–110%) for very large and bulky adenocarcinoma of left lung mass that was delivered via novel 3D MLC‐based SFRT. Due to the large patient separation, the 270° gantry angle was not used. Maximal dose to critical organs such as spinal cord (1.6 Gy), heart (4.8 Gy) and mean lung dose (1.7 Gy) was achieved. 3D, three‐dimensional; SFRT, spatially fractionated radiation therapy

**FIGURE 6:**
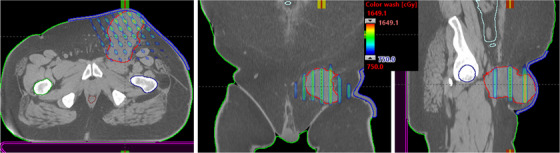
This is an example case of left enlarged pelvis lymph node of metastatic penis cancer patient treated with 3D MLC‐based SFRT. Very large pelvis lymph node that was perturbing to the skin was treated for a large single‐dose of 15 Gy with a 110% hotspot inside the GTV via 3D MLC‐based SFRT. For four of six treatment fields with 10‐MV beam, 5 mm bolus, a maximal dose rate of 600 MU/min was used. Gantry angles, 270° and 210°, were not used; Lower maximal dose to critical organs, left femoral head (4.3 Gy) and small bowel (1.3 Gy), was achieved. 3D, three‐dimensional; GTV, gross tumor volume; SFRT, spatially fractionated radiation therapy

**FIGURE 7:**
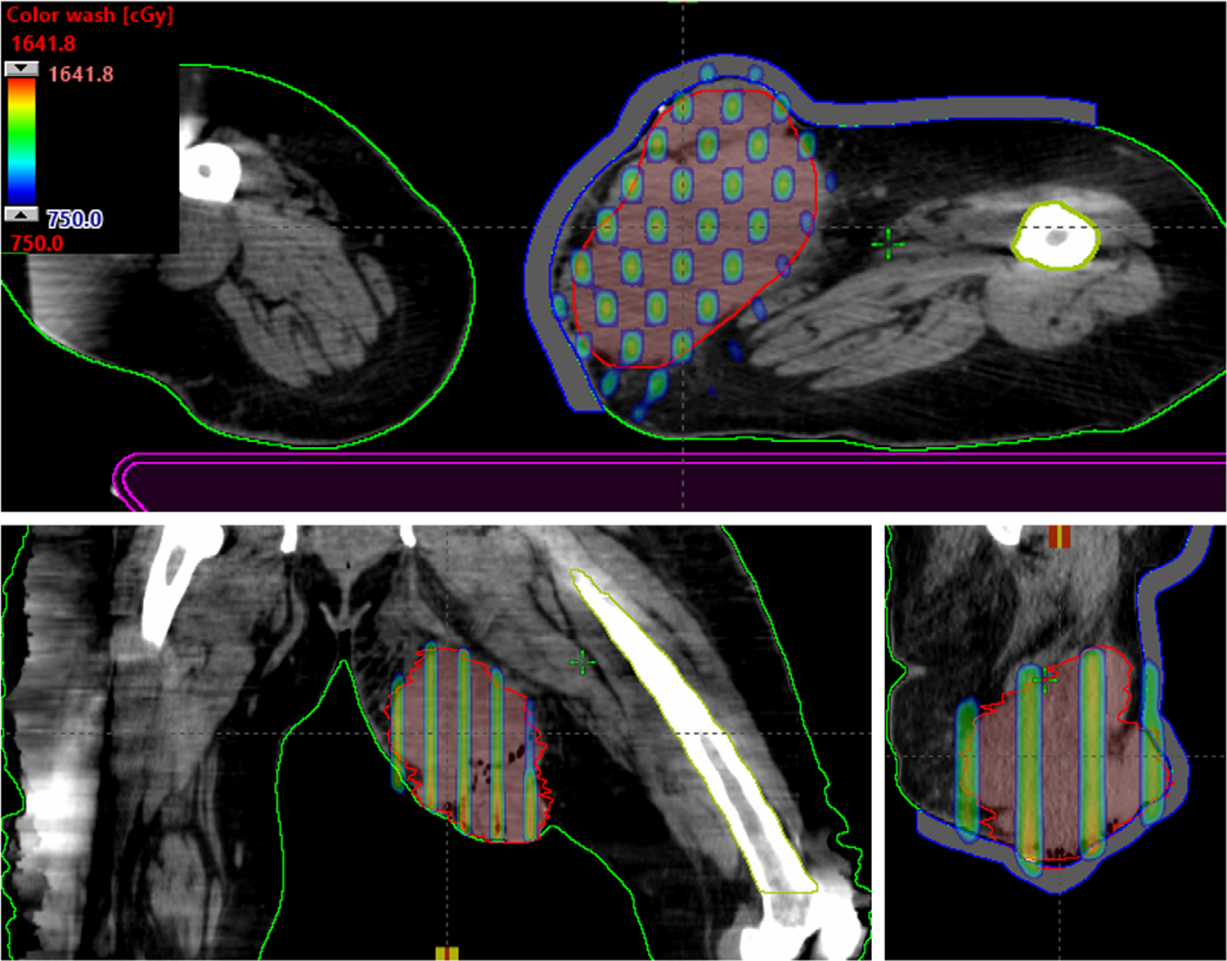
Demonstration of the axial, coronal, and sagittal views of isodose colorwash (50%–110%) for a patient treated for a bulky left upper leg soft tissue sarcoma using novel 3D MLC‐based SFRT (15 Gy in one fraction) plan. To minimize the dose of left femur and right leg, 270° and 90° beams were not used. SFRT, spatially fractionated radiation therapy

Our simple 3D‐MLC method was clinically very useful for managing large and bulky unresectable masses in any parts of the patient's anatomy (except brain), especially for deep‐seated masses. Therefore, additional site‐specific clinical example cases of SFRT treatment are presented for review in Figures 3, 4, 5, 6, 7. In Figure 3, we demonstrate the SFRT plan that was delivered for the treatment of Markel cell carcinoma of the right neck via novel MLC‐based 3D‐conformal SFRT. This large right neck mass (265 cm^3^, equivalent to 8.4‐cm diameter) was treated for a single dose of 15 Gy, with a 110% hotspot inside the GTV. The GTV (V7.5 Gy) = 53%, and a mean GTV dose of 15‐Gy prescription was 8.7 Gy. PVDR was 3.3. Maximal dose to immediately adjacent critical organs, such as spinal cord and brainstem, was 2.5 and 2.6 Gy, respectively. For five of six treatment fields with 6‐MV beam, 5 mm bolus, a maximal dose rate of 600 MU/min (90° gantry angle was not used) was used. A total of 1995 MU doses were delivered in less than 3.5 min via CBCT‐guided SFRT, with total treatment time (including patient setup, CBCT‐imaging, and verification) of less than 15 min. An additional 60 Gy therapeutic dose in 30 fractions was delivered to the right neck after administering a single dose of 15‐Gy SFRT while maintaining dose to critical organs.

A clinical example of SFRT plan that was delivered for the treatment of neglected female right breast via novel MLC‐based 3D‐conformal SFRT is shown in Figure 4. Very large right breast mass (1084 cm^3^, equivalent to 12.7‐cm diameter) was treated for a single dose of 15 Gy, with a 110% hotspot inside the GTV. The GTV (V7.5 Gy) = 52%, and a mean GTV dose of 15 Gy prescription was 8.7 Gy. PVDR was 3.3. Maximal doses to critical organs are as follows: spinal cord (1.9 Gy), left breast (1.6 Gy), heart (3.9 Gy), and mean lung dose (1.3 Gy). For four of six treatment fields with 6‐MV beam, 1‐cm bolus, a maximal dose rate of 600 MU/min (90° and 150° gantry angles were not used) was used. A total of 1935 MU doses were delivered (in <3.5 min) via image‐guided SFRT with a total treatment time (including patient setup, imaging, and verification) of within 15 min. In addition to a single dose of 15 Gy SFRT, subsequent 50 Gy therapeutic dose in 25 fractions was delivered for the treatment of the right breast.

The SFRT plan was delivered for the treatment of bulky adenocarcinoma of lung mass via novel MLC‐based 3D‐conformal SFRT (Figure 5). Very large left chest mass (846 cm^3^, equivalent to 11.7 cm diameter) was treated for a single dose of 15 Gy SFRT. The GTV(V7.5 Gy) = 51% and mean GTV dose of 15 Gy prescription was 7.8 Gy. PVDR was 3.4. Maximal doses to critical organs are as follows: spinal cord (1.6 Gy), heart (4.8 Gy), and a mean lung dose (1.7 Gy). For five of six treatment fields with 6‐MV beam, a maximal dose rate of 600 MU/min (270° gantry angle was not used) was used. A total of 1851 MU doses were delivered (in <3.5 min) via CBCT‐guided SFRT on the same day with total treatment time (including patient set up and verification) of within 15 min. Moreover, palliative 30 Gy in 10 fractions to the left chest was delivered after a single dose of 15 Gy SFRT treatment.

We have demonstrated the 3D MLC‐based SFRT plan that was delivered for the treatment of left enlarged pelvis lymph node of metastatic penis cancer patient in Figure 6. Very large pelvis LN mass that was perturbing to the skin (624 cm^3^, equivalent to 10.6‐cm diameter) was treated for a single dose of 15 Gy with a 110% hotspot in the LN GTV via MLC‐based SFRT. The GTV(V7.5 Gy) = 53%, and a mean GTV dose of 15 Gy prescription was 8.3 Gy. PVDR was 3.4. Maximal doses to critical organs are as follows: left femoral head (4.3 Gy) and small bowel (1.3 Gy). For four of six treatment fields with 10‐MV beam, 5‐mm bolus, a maximal dose rate of 600 MU/min (270° and 210° gantry angles were not used) was used. A total of 2003 MU doses were delivered (in <3.5 min) via CBCT‐guided SFRT on the same day as CT simulation, with a total treatment time (including patient setup, imaging, and verification) of less than 15 min on TrueBeam Linac. A therapeutic 57.5 Gy dose in 23 fractions treatment to the left enlarged LN PTV was delivered after a single dose of 15 Gy SFRT while respecting the dose tolerances of the critical organs, including small bowel.

Finally, Figure 7 shows the SFRT plan that was delivered for the treatment of the bulky left upper leg soft tissue sarcoma via novel MLC‐based 3D‐conformal SFRT. Very large leg sarcoma (878 cm^3^, equivalent to 11.8‐cm diameter) was treated for a single dose of 15 Gy with a 110% hotspot in the sarcoma GTV. The GTV(V7.5 Gy) = 63%, and a mean GTV dose of 15Gy prescription was 8.9 Gy. PVDR was 3.2. Maximal dose to left femur was 3.4 Gy. For four of six treatment fields with 10 MV beam, 1 cm bolus, a maximal dose rate of 600 MU/min was used. To avoid the unwanted dose to the right leg, 270° and 90° gantry angles were not used. A total of 1947‐MU doses were delivered (in <3.5 min) via image‐guided SFRT on the same day as CT simulation, with a total treatment time (including patient setup and verification) of within 15 min. In addition to 15 Gy SFRT treatment, this patient received a conventional therapeutic dose of 50 Gy in 25 fractions to the connective soft tissue sarcoma.

For all clinical cases, including example patients presented here, overall treatment planning time was typically less than an hour for an experienced planner. As mentioned previously, patient‐specific quality assurance time at the machine is not needed for these 3D‐conformal MLC‐based plans. Independent dosimetric verification of physics second check of monitor unit (MU) per treatment field can be done via in‐house TMR‐based spreadsheet calculation or commercially used second MU check software such as RadCalc QA software (LAP, LifeLine Software, Inc. Austin, TX) or MU check (Oncology Data Systems, Inc. Oklahoma City, OK) within a few minutes—enabling the same day SFRT treatment, as CT simulation.

## 4 SUMMARY AND CONCLUSION

In summary, the clinically novel and useful 3D‐MLC‐based forward planning method generates a highly nonuniform, brachytherapy sieve‐like dose distribution that can be delivered using image‐guided SFRT. The major advantages of our approach are as follows: (1) it eliminates all the major difficulties of traditional physical single‐field GRID‐block, (2) it avoids other difficulties and complexities of the modern GRID therapy approaches as described previously, and (3) this simple MLC‐based approach can easily be adopted by any radiation therapy clinic and thus can provide SFRT treatment to underserved or complex patients. Moreover, it will allow for potential tumor dose escalation, while sparing immediately adjacent critical organs, and provide all the dosimetry information needed by physicians for plan review and documentation. It also has the added benefit of providing treatment on the same day or next day after CT simulation. SFRT treatment offers a great option for cancer patients with large and bulky tumors by potentially allowing for quick debulking unresectable large tumor masses for pain relief or curative treatment and provides an opportunity for surgical resection.

Our ongoing research includes further optimizing the plan quality via spherical rather than cylindrical dose distribution by scripting and automating the template plan in the Eclipse TPS. Exploiting immunotherapy with our simple, yet clinically useful SFRT approach in the management of large and bulky tumors merits future investigation.^11^, ^12^ Additionally, further reducing dose to adjacent critical organs, while escalating tumor dose to the deep‐seated bulky masses by delivering even faster SFRT treatment with FFF‐beams,^23^ and investigating its radiobiological response and tumor motion management^25^ via DIBH for 3D‐conformal MLC‐based SFRT patients are currently underway. Moreover, a clinical follow‐up of patients in a prospective clinical trial with higher tumor dose to deep‐seated bulky tumors is ongoing to evaluate the pain‐relief, tumor local control rates, and treatment‐related toxicity profile for patients with MLC‐based SFRT. We recommend other centers to use this MLC‐fitting algorithm for large and bulky tumors to expand the access of pain‐relief to patients and/or curative treatment of SFRT to the underserved patient cohort.

## CONFLICT OF INTEREST

All coauthors have no conflict of interest to declare.

## FUNDING INFORMATION

None.

## AUTHOR CONTRIBUTIONS

Damodar Pokhrel, PhD, designed the 3D MLC‐based SFRT treatment method and MLC‐fitting algorithm, collected data, and drafted the preliminary manuscript. Mark E. Bernard, MD, William St. Clair, MD, PhD, and Mahesh Kudrimoti, MD, provided radiation oncology clinical expertise and supervision of the paper. Richard Mallory, BS, assisted the literature review and helped in drafting the manuscript. All coauthors revised, edited, and approved the final submission of the manuscript.

## Data Availability

All data are available on request due to privacy/ethical restrictions.
